# Intentions and attitudes of caregivers towards enrolment of their children and adolescents living with HIV into remission trials involving analytic treatment interruption

**DOI:** 10.1002/jia2.70084

**Published:** 2026-02-10

**Authors:** Holly L. Peay, Kennedy Otwombe, Shaun Barnabas, Ana Barrios‐Tascon, Maria Grazia Lain, Tacilta Nhampossa, Diana Rutebarika, Thidarat Jupimai, Moherndran Archary, Almoustapha‐Issiaka Maiga, Avy Violari, Moira J. Spyer, Kavidha Reddy, Paolo Palma, Savita Pahwa, Mathias Lichterfeld, Mark Cotton, Louise Kuhn

**Affiliations:** ^1^ RTI International Research Triangle Park Durham North Carolina USA; ^2^ Faegre Drinker Consulting Washington DC USA; ^3^ Perinatal HIV Research Unit, Chris Hani Baragwanath Academic Hospital; and School of Public Health, Faculty of Health Sciences University of the Witwatersrand Johannesburg South Africa; ^4^ FAMCRU, Tygerberg Academic Hospital Stellenbosch University Cape Town South Africa; ^5^ Gertrude H. Sergievsky Center, Vagelos College of Physicians and Surgeons Columbia University Irving Medical Center New York New York USA; ^6^ Fundação Ariel Glaser contra o SIDA Pediátrico Maputo Mozambique; ^7^ Manhiça Health Research Center Manhiça Mozambique; ^8^ Joint Clinical Research Centre Kampala Uganda; ^9^ Center of Excellence for Pediatric Infectious Diseases and Vaccines, Faculty of Medicine Chulalongkorn University Bangkok Thailand; ^10^ Africa Health Research Institute Durban South Africa; ^11^ Centre Hospitalier Universitaire Gabriel Touré University of Sciences Techniques and Technologies of Bamako Bamako Mali; ^12^ UCL Great Ormond Street Institute of Child Health London UK; ^13^ Research Unit of Clinical Immunology and Vaccinology Bambino Gesù Children's Hospital Rome Italy; ^14^ University of Miami Miami Florida USA; ^15^ Ragon Institute Harvard University Cambridge Massachusetts USA

**Keywords:** analytic treatment interruption trials, children and adolescents with HIV, HIV remission, knowledge and attitudes, parents and guardians of children living with HIV, vignette studies

## Abstract

**Introduction:**

The development of approaches and interventions to achieve HIV remission continues to accelerate. Children living with HIV who started antiretroviral therapy (ART) at a young age and sustain viral suppression are an ideal clinical trial population. Trials may require analytic treatment interruptions (ATIs). Paediatric trials depend on the willingness of guardians to consent to child participation, yet there are few data about guardian willingness or attitudes. Here, we investigated the opinions of guardians of children likely to be eligible for ATI trials.

**Methods:**

Children and youth who started ART ≤ 3 months of age, and who remained well‐controlled on ART older than 7 years, were recruited in South Africa, Mozambique, Uganda, Mali and Thailand. A survey was conducted among guardians of these paediatric participants. The survey utilized a vignette describing a trial with ATI and assessed attitudes and intentions (measured on 7‐point scales) of the guardians regarding their children's participation in a hypothetical trial.

**Results:**

Guardians of 99 children were recruited. Guardians’ median age was 45 years (range 24–73) and most (89.9%) were female. The median age of the child or youth with HIV was 13.2 years (range 7–18.5 years). Most respondents endorsed a positive intention to enrol their child in a future HIV remission trial (mean 6.5 [SD:1.3] on a 7‐point scale), with significant variation across the sites (*p* = 0.0024). Most respondents strongly endorsed a range of trial benefits, including better future HIV treatments (93.8%) and access to better care (88.0%). Some endorsed concern about the trial burden to themselves (33.3%) and the child (35.4%). Almost half strongly believed that the trial would result in the child no longer needing ART (48%) and the child being cured of HIV (46.5%).

**Conclusions:**

Across multiple countries, guardians of children and youth who were treated early were positive about participation in trials with ATI. Only a third expressed some concern about trial burden, while almost half had unrealistic expectations about potential benefits. Recruitment into trials involving ATI will need to include effective communication strategies to ensure that participants and caregivers are adequately informed about burden, potential risks and the likelihood of personal benefit.

## 1 INTRODUCTION

HIV remission research aims to develop approaches that either eliminate HIV from the body or, more feasibly, control HIV so that long‐term antiretroviral therapy (ART) is no longer needed [1]. Approaches and interventions to achieve HIV remission continue to accelerate [1]. Children living with HIV who started ART at young ages and who sustain viral suppression are an ideal clinical trial population [2, 3]. This is because early treatment reduces replication‐competent virus in reservoirs and supports development of anti‐HIV immune responses [2, 3]. Many HIV remission trials will require analytic treatment interruptions (ATIs), which, although an accepted design for persons over the age of 2 years, have associated risks [4]. Guidelines for the ethical design and conduct of paediatric remission trials, including safety monitoring, inclusion and exclusion criteria, and other features, have been published [4].

Paediatric HIV trials depend on the willingness of an adult surrogate to consent to child participation, yet there are few studies of parent or guardian attitudes. In a review article summarizing studies on perceptions and ethical issues in adolescent HIV research participation, five studies of parents or guardians and 19 studies of adolescents are reported; they pertained to attitudes about prevention, surveillance and vaccine studies [5]. Overall, there was interest in and positive attitudes about HIV research, yet studies described concerns about privacy and confidentiality, stigma, research‐related risks and the need for transparency in the consent process. Motivators included altruism, potential personal benefit, increased access to healthcare, improved education, trust in research teams and compensation [5].

Subsequent qualitative research conducted in Kenya explored participation in longitudinal clinical HIV research among youth living with HIV and caregivers [6]. Concerns included accidental disclosure of HIV status, stigma, negative mental health effects, risks of blood draws, side effects from an experimental treatment, intimidation by researchers and the potential coercive nature of study compensation. Possible benefits included altruistic benefits, improved medication adherence, better clinical care, learning about HIV, positive psychosocial benefits and financial compensation [6].

There is a larger body of research among adults living with HIV. A review identified 78 social/behavioural articles about HIV remission trials among adults living with HIV [7]. Overall, positive attitudes about the importance of remission research and relatively high intentions to participate were found, with most studies reporting willingness to participate >50%. Barriers included medical risks and side effects, social risks such as HIV transmission, and practical considerations, including trial burden. Facilitators were altruism, perceived personal benefits, personal experiences and beliefs, and study conditions that limit risks or barriers to participation. Most papers were from the United States and other high‐income countries [7].

Several studies published subsequent to the review reported on adults’ attitudes towards remission trials that include ATI. A qualitative study in Durban, South Africa, evaluated opinions of community respondents (with mixed HIV status) and found positive attitudes stemming from the potential to mitigate ART cost, address adherence challenges or eradicate HIV [8]. Psychosocial benefits of optimism and stigma reduction were reported. Challenges included meaningful education and informed choice, risks and uncertainty associated with the ATI, risks associated with the experimental agent, confidentiality risks and misinformation [8]. Another study among young women living with HIV in Durban reported positive attitudes about remission trials [9]. Reported barriers were potential health risks, HIV stigma and the potential for HIV transmission during an ATI. Facilitators to participation were trust in the research team and close monitoring during the trial [9]. A study in Soweto, South Africa, among adults living with HIV found moderate acceptability of cure‐related interventions. Acceptability was contingent on the expected efficacy and safety of the interventions [10].

Though ethical challenges exist, children living with HIV have the greatest likelihood of benefiting from remission trials. The limited data do not support specific risks from ATI to children [4]. Yet, we found no published studies specifically assessing the attitudes of guardians about enrolling children in HIV remission trials. Data are particularly needed from low‐ and middle‐income countries (LMICs) where paediatric HIV burden is highest and individual‐level and structural burdens to trial participation are challenging.

To address this gap, we investigated attitudes and intentions of parents or legal guardians of children likely to be eligible for ATI trials, with the objective of assessing the feasibility and acceptability of future paediatric HIV remission trials conducted in LMICs.

Our exploratory aims were to:

1. Characterize structural, family and individual‐level factors that may impact inclusion in future trials.
2. Describe guardians’ intentions to enrol their child in future HIV remission trials and assess for differences in intentions based on trial site.
3. Describe guardians’ attitudes towards future HIV remission trials and perceived behavioural control/barriers and facilitators to participation.

Our overall objective was to strengthen the design of future trials in similar populations, including educational and counselling materials that support authentic informed consent.

## 2 METHODS

The Child and Adolescent Research Moving towards Alternative Interventions Globally (CARMA‐GLOBAL) study recruited 99 children and adolescents living with HIV between 10 November 2022 and 19 December 2023. The study was conducted at eight sites: Tygerberg Hospital (Cape Town, South Africa), Chris Hani Baragwanath Hospital (Soweto, South Africa), Africa Health Research Institute (Durban, South Africa), Fundação Ariel Glaser contra o SIDA (Maputo, Mozambique), Manhiça Health Research Center (Manhiça, Mozambique), Joint Clinical Research Center (Kampala, Uganda), Centre Hospitalier Universitaire Gabriel Touré (Bamako, Mali) and Center of Excellence for Pediatric Infectious Diseases and Vaccines, Faculty of Medicine, Chulalongkorn University (Bangkok, Thailand).

The protocol was approved by the Institutional Review Boards (IRB) of each site. The study conformed to principles in the Declaration of Helsinki and the US Federal Policy for the Protection of Human Subjects. Written informed consent was provided by a parent or legal guardian, and assent was provided by minors where appropriate, based on local guidelines.

Researchers at each site reviewed records to select children and youth living with HIV who had started ART ≤3 months of age, were at least 7 years of age, currently in care and virally suppressed on ART (2 or more viral load measurements <50 copies/ml in the past 2 years) and, based on past viral load results, *expected* to have undetectable viral load when tested at enrolment. Other exclusion criteria included current tuberculosis treatment, hepatitis B or C infection, severe neurocognitive impairment, current pregnancy, malignancy or the use of immunosuppressive therapy.

Parents or legal guardians of the paediatric participants were administered a survey that included a vignette of a remission trial with ATI to assess their attitudes and intentions towards such a trial for their children. The survey design was informed by the Theory of Planned Behavior (TPB) [11], which posits that an individual's behaviour is determined by their intention to engage in the behaviour. This intention results from the individual's attitudes, beliefs about others’ approval or disapproval, and perceived behavioural control in the presence of barriers and facilitators to the action. The TPB was used by the first author (HLP) to generate a first draft of the instrument and the supporting vignette. The instrument was reviewed by study teams at all sites and then revised by the first (HLP) and senior (LK) authors based on this feedback. Investigators at all sites reviewed the final version.

All items were measured using semantic differential scales (Likert‐type scale scored 1−7), and negatively oriented items were reverse‐scored. Higher scores indicate higher intentions or more positive attitudes. Response options were colour‐coded to support respondent understanding. All study materials were translated into local site language(s) using professional translation services with back‐translation.

**Figure 1:**
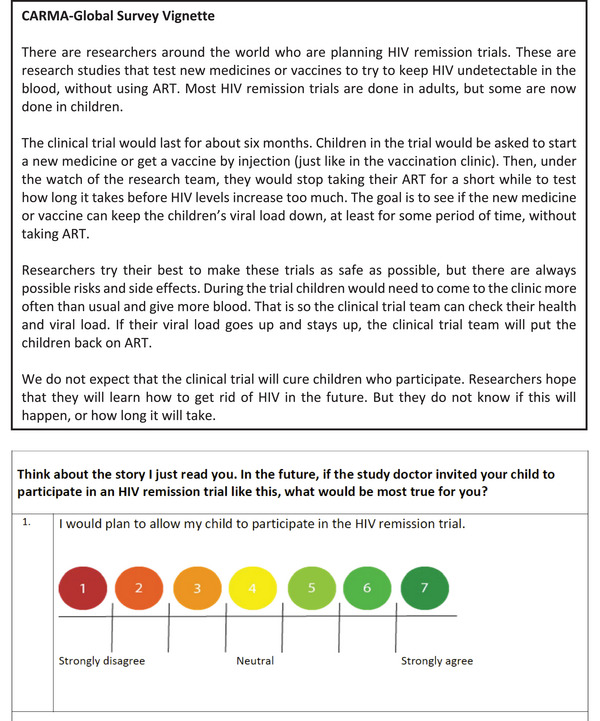
Vignette and example item.

Surveys were administered verbally. The first author trained study teams to read the vignette word‐for‐word and ask each survey question exactly as written, and guide respondents to select a response while showing the colour‐coded response scale (Figure 1) for each item. The study teams were trained not to elaborate on the text, and if asked for clarification, to re‐read relevant sections. The intention was to anchor responses to the specific brief vignette.

The attitude survey comprised the following domains:

### 2.1 Trial participation intention

Intention was rated with 1 item related to enrolling the child in the remission trial (“I would plan to allow my child to participate in the HIV remission trial”).

### 2.2 Trial attitudes

The survey incorporated 7 trial attitude items with two domains: trial‐related (e.g. “HIV remission trials are a good idea”) and child‐related (e.g. “My child participating in an HIV remission trial would be helpful”).

### 2.3 Benefit attitudes, direct benefit to child

Two items specifically assessed the over‐estimate of benefit to the child (e.g. “My child would be cured of HIV because of the trial”).

### 2.4 Benefit attitudes, altruistic and ancillary benefits

Four items assessed ancillary benefits ranging from altruistic benefits to improved healthcare (e.g. “I would get a better understanding of my child's HIV”).

### 2.5 Perceived control/barriers and facilitators

Eight items measured decision‐making control, facilitators and barriers/burden (e.g. “Whether or not I allow my child to participate in the HIV remission trial is up to me” and “It would be too much effort for my child”).

### 2.6 Other's approval

Three items measured perceptions of others’ willingness/approval regarding HIV remission trials (e.g. “My child's doctors would think that my child should participate in the HIV remission trial”).

Other social and household characteristics were collected through a structured interview with the guardian. Items included the respondent and child's age and clinical history, household composition, indicators of socio‐economic status and information access, and education. Data were collected on whether the child had been informed of his or her HIV status, the age at which the disclosure occurred and the person responsible for the disclosure. Clinical records were reviewed to determine the age of confirmation of HIV status, age of ART start, regimen changes and interruptions, and prior laboratory results and clinical events. Data were entered into a centralized REDCap database.

Descriptive analyses were conducted (frequencies, means, medians, interquartile range). Comparisons across sites were by Kruskal−Wallis tests with pairwise comparisons using Mann−Whitney U tests. Planned analysis to assess factors association with intention to participate could not be conducted due to low variability in guardian responses to the intention item. All analyses were conducted with SAS software, version 9.4 (Cary, NC, USA).

## 3 RESULTS

Ninety‐nine guardians of children and youth living with HIV were recruited. Guardians’ median age was 45 years (range 24–73) and almost all (89.9%) were female. The median age of the child or youth was 13.2 years (range 7–18.5). Median age of ART initiation was 2 months.

Table 1 shows site differences in child and respondent characteristics, including age of child and respondent type, educational attainment and employment. A deceased father was reported for 29.3% of children, and 14.3% had a deceased mother. Additionally, 49.2% of fathers did not reside in the household. There was variability in markers of socio‐economic status, including living in homes with an indoor toilet, water, electricity, internet and computers. Overall, nearly a quarter (22.2%) reported experiencing hunger at school due to food insecurity at home. Approximately half of children (52.5%) had their HIV status disclosed, with a median disclosure age of 12 years (range 3–16 years). When stratified by age, full disclosure had taken place among 89.8% of youth ≥13 years (4.1% partial disclosure), 16% of children 9–12 years (44% partial disclosure) and 11.8% of children ≤8 years. Adults living in the same household and those in the same school knew their HIV status in 33.7% and 17.2%, respectively.

**Table 1:** Characteristics of the children and youth living with HIV and their guardians, household and social factors and disclosure profile by site

Site *N*	South Africa Cape Town 17	South Africa Soweto 15	South Africa Durban 9	Mozambique Maputo 8	Mozambique Manhiça 8	Uganda Kampala 16	Mali Bamako 12	Thailand Bangkok 14	Total 99
**Child's age at enrolment (years)**									
Median (p25−p75)	17.3 (16.8−17.6)	16.3 (15.9−16.8)	11.8 (10.6−13.3)	9.6 (7.3−13.0)	8.3 (7.3−10.2)	11.6 (10.4−13.8)	11.8 (11.2−15.8)	8.3 (7.8−10.5)	13.2 (9.6−16.7)
Min	15.5	7	8.3	7	7.2	7.9	10.3	7.4	7
Max	18	18.1	16.7	13.4	11.1	17	18.5	16.7	18.5
**Age at ART start (months)**									
Median (p25−p75)	1 (1−1)	2 (1−2)	2 (1−3)	2 (1−3)	2 (2−2.5)	2 (1−2)	3 (1−3)	2 (1−2)	2 (1−2)
**Time on ART (years)**									
Median (p25−p75)	17.2 (16.7−17.4)	16.2 (15.7−16.6)	11.5 (10.3−13.1)	9.5 (7.2−12.7)	8.1 (7.1−10)	11.4 (10.2−13.8)	11.3 (10.8−11.5)	8.1 (7.4−10.2)	12.1 (9−16.4)
Min	15.3	7	8.3	6.9	6.9	7.8	10.2	7.3	6.9
Max	17.9	17.9	16.6	13.3	10.9	16.7	11.5	16.4	17.9
**Caregiver respondent (*N*, %)**									
Biol. mother	14 (82.3)	14 (93.3)	3 (33.3)	5 (62.5)	6 (75)	13 (81.3)	8 (66.7)	2 (14.3)	65 (65.7)
Grandmother	1 (5.9)	0	1 (11.1)	2 (25)	2 (25)	0	1 (8.3)	10 (71.4)	17 (17.2)
Father	1 (5.9)	0	2 (22.2)	1 (12.5)	0	1 (6.3)	2 (16.7)	1 (7.1)	8 (8.1)
Aunt	0	1 (7.1)	2 (22.2)	0	0	1 (6.3)	0	0	4 (4.0)
Grandfather	0	0	0	0	0	0	1 (8.3)	1 (7.1)	2 (2.0)
Other	1 (5.9)	0	1 (11.1)	0	0	1 (6.3)	0	0	3 (3.0)
**Caregiver's age (years)**									
Median (p25−p75)	46 (42−50)	44 (43−53)	46 (33−52)	41.5 (38−54)	39 (34.5−47.5)	40 (32.5−43)	49 (43−65)	58.5 (48−63)	45 (40−53)
Min	40	40	24	37	31	26	42	29	24
Max	56	64	61	63	73	63	70	70	73
**Highest grade the primary caregiver finished at school**									
Median (p25−p75)	9 (8−12)	11 (11−12)	12 (12−12)	5 (5−7)	12 (*n* = 1)	10.5 (7−11)	13 (13−13)	6 (6−9)	11 (7−12)
Min	4	9	9	2		4	13	4	2
Max	12	13	13	13		13	13	13	13
**Caregiver has a paid job?**									
Yes	7 (41.2)	4 (26.7)	3 (33.3)	1 (12.5)	4 (50)	12 (75)	1 (8.3)	11 (78.6)	43 (43.4)
No	8 (47.1)	11 (73.3)	6 (66.7)	7 (87.5)	4 (50)	4 (25)	11 (91.7)	3 (21.4)	54 (54.6)
Unknown	2 (11.8)	0	0	0	0	0	0	0	2 (2.02)
**Child's biological father alive?**									
Yes	8 (47.1)	10 (66.7)	5 (55.6)	5 (62.5)	4 (50)	13 (81.3)	7 (58.3)	10 (71.4)	62 (62.6)
No	4 (23.5)	5 (33.3)	3 (33.3)	3 (37.5)	4 (50)	2 (12.5)	5 (41.7)	3 (21.4)	29 (29.3)
Don't know	5 (29.4)	0	1 (11.1)	0	0	1 (6.2)	0	1 (7.1)	8 (8.1)
**Child's biological mother alive?**									
Yes	14 (82.4)	14 (93.3)	7 (77.8)	7 (87.5)	8 (100)	14 (93.3)	9 (75)	9 (64.3)	82 (83.7)
No	3 (17.7)	1 (6.7)	2 (22.2)	1 (12.5)	0	0	2 (16.7)	5 (35.7)	14 (14.3)
Don't know	0	0	0	0	0	1 (6.7)	1 (8.3)	0	2 (2)
**Tap inside the house**									
Yes	16 (94.1)	8 (53.3)	7 (77.8)	6 (75)	1 (12.5)	5 (31.3)	7 (58.3)	14 (100)	64 (64.6)
No	1 (5.9)	7 (46.7)	5 (22.2)	2 (25)	7 (87.5)	11 (68.7)	5 (41.7)	0	35 (35.4)
**Toilet inside the house**									
Yes	15 (88.2)	8 (53.3)	6 (66.7)	3 (37.5)	1 (12.5)	4 (25)	12 (100)	14 (100)	63 (63.6)
No	2 (11.8)	7 (46.7)	3 (33.3)	5 (62.5)	7 (87.5)	12 (75)	0	0	36 (36.4)
**Electricity in the house**									
Yes	17 (100)	15 (100)	9 (100)	6 (75)	4 (50)	14 (87.5)	10 (83.3)	14 (100)	89 (89.9)
No	0	0	0	2 (25)	4 (50)	2 (12.5)	2 (16.7)	0	10 (10.1)
**Computer in the house**									
Yes	3 (17.7)	6 (40)	2 (22.2)	1 (12.5)	1 (12.5)	4 (25)	3 (25)	6 (42.9)	26 (26.3)
No	14 (82.3)	9 (60)	7 (77.8)	7 (87.5)	7 (87.5)	12 (75)	9 (75)	8 (57.1)	73 (73.7)
**Internet in the house**									
Yes	1 (5.9)	13 (86.7)	6 (66.7)	4 (50)	2 (25)	13 (81.3)	4 (33.3)	14 (100)	57 (57.6)
No	16 (94.1)	2 (13.3)	3 (33.3)	4 (50)	6 (75)	3 (18.7)	8 (66.7)	0	42 (42.4)
**Sometimes have to go hungry to school because there is not enough food to eat at home?**									
Yes	1 (5.9)	4 (26.7)	4 (44.4)	3 (37.5)	3 (37.5)	4 (25)	3 (25)	0	22 (22.2)
No	16 (94.1)	11 (73.3)	5 (55.6)	5 (62.5)	5 (62.5)	12 (75)	9 (75)	14 (100)	77 (77.8)
**Does the child take an active role in taking his/her medications?**									
Yes No	16 (94.1) 1 (5.9)	15 (100) 0	8 (88.9) 1 (11.1)	5 (71.4) 2 (28.6)	8 (100) 0	11 (68.8) 5 (31.2)	11 (91.7) 1 (8.3)	12 (85.7) 2 (14.3)	86 (87.9) 12 (12.1)
**Disclosure of HIV status to the child with HIV**									
Full	17 (100)	14 (93.3)	4 (44.4)	2 (25)	1 (12.5)	5 (31.2)	6 (50)	3 (21.4)	52 (52.5)
Partial	0	0	0	3 (37.5)	0	5 (31.2)	5 (41.7)	0	13 (13.1)
Limited or no	0	0	5 (55.6)	3 (37.5)	7 (87.5)	5 (31.2)	1 (8.3)	7 (50)	28 (28.3)
Unknown	0	1 (6.7)	0	0	0	1 (6.3)	0	4 (28.6)	6 (6.1)
**If disclosure, age at disclosure (years)**									
Median (p25−p75)	13 (12−14)	13 (9−14)	12 (12−14)	10.5 (9−12)	10 (*n* = 1)	10 (6−13)	12 (12−12)	10 (8−12)	12 (10−13)
Min	7	8	12	9		3	12	8	3
Max	16	14	14	12		14	12	12	16
**Are there adults in the household where the child lives who do not know the child's HIV status?**									
Yes	14 (82.4)	13 (92.9)	7 (77.8)	1 (12.5)	6 (75)	9 (56.3)	4 (33.3)	11 (78.6)	65 (66.3)
No	3 (17.6)	1 (7.1)	2 (22.2)	7 (87.5)	2 (25)	7 (43.7)	8 (66.7)	3 (21.4)	33 (33.7)
**Someone at school know the child's HIV status?**									
Yes	1 (5.9)	3 (20)	2 (22.2)	0	5 (62.5)	4 (25)	0	02 (14.3)	17 (17.2)
No	14 (82.4)	10 (66.7)	5 (55.6)	8 (100)	3 (37.5)	12 (75)	12 (100)	12 (85.7)	76 (76.8)
Don't know	2 (11.7)	2 (13.3)	2 (22.2)	0	0	0	0	0	6 (6)

**Figure 2:**
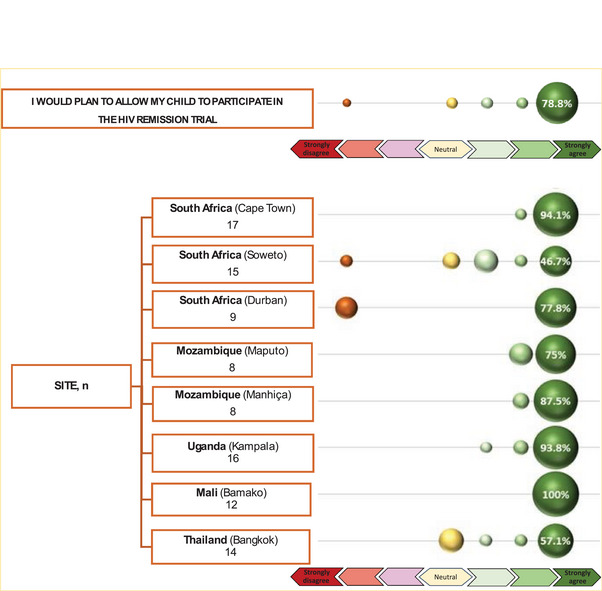
Bubble figure of intentions to enrol child in future HIV remission trials, by site.

Across all sites, most respondents endorsed a positive intention (mean 6.5 on a 7‐point scale) to enrol the child in a future HIV remission trial (Table 2 and Figure 2), corresponding to “strongly agree” ratings ranging from 100% to 46.7% across the eight sites. There were significant differences regarding intentions to enrol the child (*p* = 0.0024). After analysing each site independently, South Africa (Soweto) and Thailand (Bangkok) were significantly different from all other sites combined (*p* = 0.0009 and *p* = 0.0259), reporting less positive mean intentions (Table 2). Yet for both sites, approximately 50% of caregivers still reported strongly agreeing with the intention to enrol (Figure 2).

**Table 2:** Guardians’ intentions to enrol their child in a future HIV remission trial by site

INTENTIONS										
Site *N*	South Africa Cape Town 17	South Africa Soweto 15	South Africa Durban 9	Mozambique Maputo 8	Mozambique Manhiça 8	Uganda Kampala 16	Mali Bamako 12	Thailand Bangkok 14	*p* value	Total 99
**I would plan to allow my child to participate in the HIV remission trial**.										
1 Strongly disagree	0	1 (6.67)	2 (22.2)	0	0	0	0	0		3 (3.03)
2	0	0	0	0	0	0	0	0		0
3	0	0	0	0	0	0	0	0		0
4 Neutral	0	2 (13.33)	0	0	0	0	0	4 (28.57)		6 (6.06)
5	0	4 (26.67)	0	0	0	1 (6.25)	0	1 (7.14)		6 (6.06)
6	1 (5.88)	1 (6.67)	0	2 (25)	1 (12.5)	0	0	1 (7.14)		6 (6.06)
7 Strongly agree	16(94.12)	7 (46.67)	7 (77.78)	6 (75)	7 (87.5)	15 (93.75)	12 (100)	8 (57.14)		78 (78.79)
**Mean, SD**	6.94 (0.24)	5.6 (1.72)	5.67 (2.65)	6.75 (0.46)	6.88 (0.35)	6.88 (0.5)	7 (0)	5.93 (1.38)	0.0024	6.45 (1.29)

**Figure 3:**
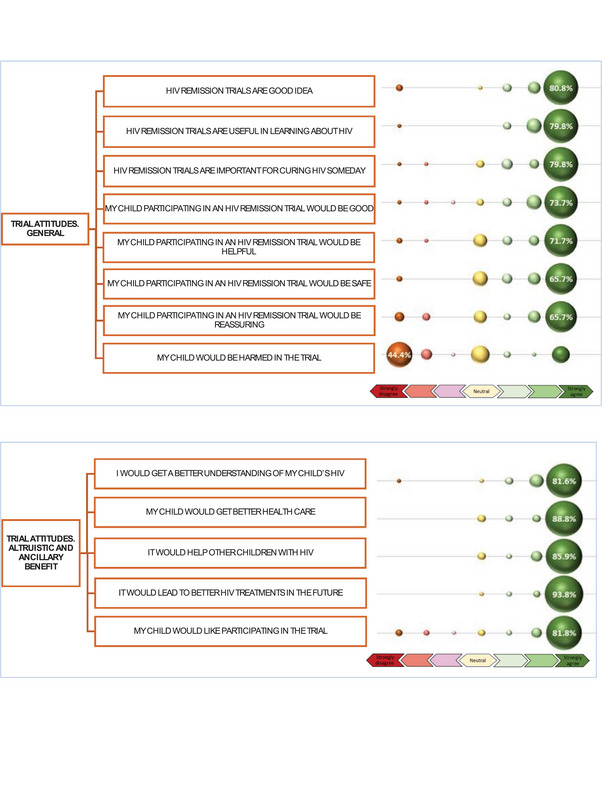
Bubble figure of (A) attitudes about HIV remission trials and (B) anticipated altruistic and ancillary benefits.

**Figure 4:**
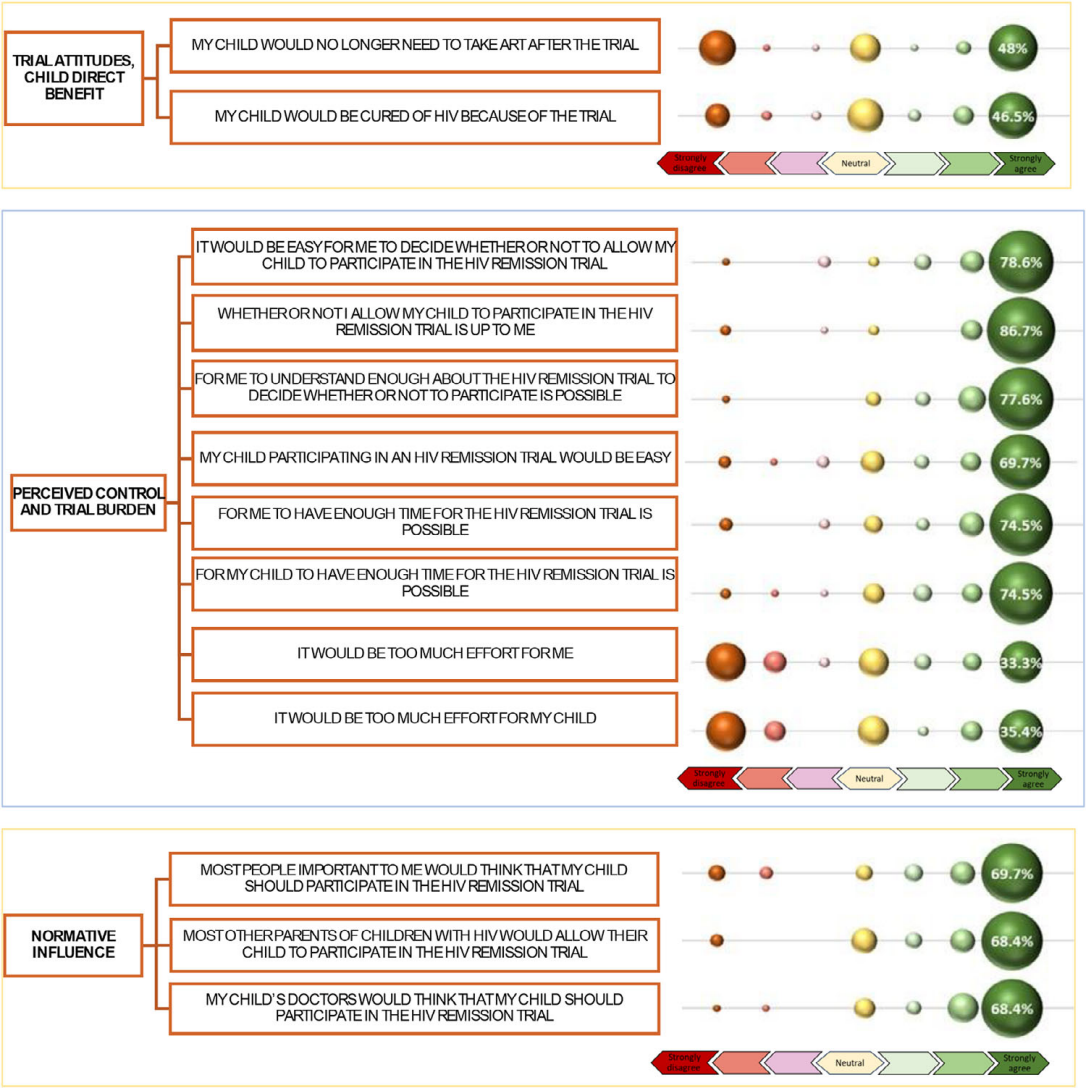
Bubble figure of (A) anticipated direct benefit to child, (B) perceived trial decision‐making control and anticipated trial burden and (C) perceptions of others’ trial attitudes.

Most guardians strongly agreed that remission trials were a good idea (80.8%), useful in learning about HIV (79.8%) and important for leading to a future cure (79.8%). Most strongly agreed that “My child participating in an HIV remission trial would be good” (73.7%) and “My child participating… would be helpful” (71.7%). Most noted that the child's participation would be safe (65.7%) and reassuring (65.7%). There was a broader distribution regarding potential for child harm in the trial, with the largest group (44.4%) endorsing that they strongly disagree that the child would be harmed (Figure 3A). Most strongly agreed that participating would lead to better future HIV treatments (93.8%), provide the child with access to better healthcare (88.8%), help other children with HIV (85.9%), provide a better understanding of the child's HIV (81.6%) and that the child would like participating (81.6%) (Figure 3B). For items assessing anticipated (over‐estimated) direct benefit to the child, almost half strongly agreed that the trial would result in the child no longer needing ART (48%) and the child being cured of HIV (46.5%) (Figure 4A).

For items about decision‐making control, most respondents strongly agreed that participation choice would be up to them (86.7%), that it would be easy for them to decide whether the child could participate (78.6%) and that they can understand sufficiently to make a decision (77.6%). When asked about trial‐related barriers, 35.4% strongly agreed that participation would be “too much effort” for the child, and 33.3% strongly agreed that it would be “too much burden” for the respondent. Most strongly agreed that they (74.5%) and their child (74.5%) had enough time for trial participation. Most (69.7%) strongly agreed that participation in the HIV remission trial would be easy (Figure 4B).

When asked about perceptions of others’ approval, most respondents strongly agreed that people important to them would think the child should participate (69.7%), the child's doctors would think the child should participate (68.4%) and that most other parents of children with HIV would allow their children to participate (68.4%) (Figure 4C).

## 4 DISCUSSION

We identified and engaged caregivers of a relatively large, multi‐LMIC cohort of children with viral load below detection at the time of enrolment in this study. These are the type of children likely to be recruited for future HIV remission trials. After listening to a vignette about an HIV remission trial, most caregivers endorsed a positive intention to enrol their child in the future trial (mean of 6.5 on a 7‐point scale). Though caregivers from Soweto in South Africa and Bangkok had significantly lower intentions compared to all other sites, the mean intention ratings at both sites were still positive towards participation. Thus, when anchored to the trial described in the vignette, there were positive intentions to participate among most caregivers at all sites.

Similarly, most caregivers reported positive attitudes about HIV remission trials, endorsed positive outcomes to advance science and improve treatment options, and endorsed the potential for ancillary benefits to the child in terms of better HIV care and improved knowledge of the child's HIV. Caregivers anticipated being able to make an autonomous choice about trial participation. Most perceived that others (including people close to them and their child's doctor) would endorse trial participation. Although the vignette described a 6‐month trial with frequent clinic visits and regular blood draws, most caregivers reported the feasibility of participation for themselves and their child. Though this study is testing a hypothetical, these results are optimistic in terms of the likely success of enrolment into future HIV remission trials.

We intentionally included two items about over‐estimation of potential trial benefits. Even with this language in the vignette, “We do not expect that the clinical trial will cure children who participate. Researchers hope that they will learn how to get rid of HIV in the future. But they do not know if this will happen, or how long it will take,” almost half strongly agreed that “My child would be cured of HIV because of the trial.” In addition, though the vignette included this statement, “Researchers try their best to make these trials as safe as possible, but there are always possible risks and side effects,” most noted that the child's participation would be safe and reassuring. Only a minority agreed with the concern that the child would be harmed in the trial. Despite the positive endorsement of enrolling, a third believed there was “too much effort” involved. It is interesting, given the time commitments and burdens of many trials, that this percentage is so low, particularly given high levels of resource constraints; this finding may be partially explained by limitations associated with a hypothetical study. Future trials should carefully consider how trial‐related burdens can be mitigated.

While providing positive indicators of parent attitudes and willingness to participate, this study reflects concerns about HIV remission trials previously reported in the adult literature [7] and literature about adolescent participation in HIV clinical research [5]: the need for tailored education and a careful, participant‐focused informed consent (age‐appropriate assent) process. We acknowledge that our brief hypothetical vignette is not nearly as comprehensive as a trial‐informed consent and may have contributed to participant attitudes concerning trial outcomes and risks. Nevertheless, our data identify critical areas where trialists need to focus their attention on educating caregivers and adolescents, including the anticipated lack of individual benefit, the chance for side effects and serious adverse events, and the trial protocol burden. Similar to some other data from adults with HIV [12, 13], our finding of overestimation of trial‐related health benefits likely reflects optimism for better future treatments, yet also suggests a pervasive need for careful attention to decision support and robust informed consent processes to counteract knowledge gaps and therapeutic misestimation or misconception [13]. Efforts to improve understanding and informed choice must be tailored to relevant populations. In our case, most respondents did not have a computer in the home, over 40% had no internet in the home and many respondents had relatively low educational attainment. These practical barriers must also be considered in education‐based interventions.

Furthermore, only slightly more than half of the children had their HIV status fully disclosed, and for more than half, there was one or more adult in the household unaware of the child's status. Incomplete disclosure raises practical and ethical challenges to paediatric HIV research, and poor parental communication and disclosure have been identified as barriers to HIV research participation in adolescents [14]. It is reassuring that among adolescents ≥13 years in our study, 90% had undergone full disclosure. However, for those 7–12 years, full disclosure was only 14%. At the 2nd Consensus Workshop on Analytical Treatment Interruption in HIV Research Trials held in Nairobi in May 2024, full disclosure was considered optimal for potential participants aged 7–17 years to assent [15]. It was acknowledged that, if acceptable to local ethics committees, pre‐adolescent children (7–12 years) whose HIV status has not yet been fully disclosed could receive a separate assent form not mentioning HIV. It was also agreed that any adolescents older than 12 years whose status was not yet disclosed should be placed on an accelerated disclosure pathway [15]. For sexually active adolescents, full disclosure is required to provide assent to a trial involving ATI. Additional data are needed regarding remission trial preferences for consent and assent among adolescents and young adults living with HIV; some may believe they are appropriate independent decision‐makers [14], though there may be comprehension and maturity challenges in adolescents under 18 [16]. Rates of disclosure vary greatly across settings and are related to individual child and family factors as well as practices within the health service [17, 18].

### 4.1 Limitations

This study is limited by relatively small sample sizes at each clinical site. The results may reflect positive response bias (unwillingness of participants to provide responses perceived as negative) and possibly selection bias (over‐representing more willing respondents). Hypothetical studies may overestimate willingness and may not reflect actual participation.

Guardian attitudes were in direct response to a brief remission trial vignette, and perceptions and willingness must be interpreted in light of the trial characteristics provided. The brevity and lack of specifics may have contributed to guardian attitudes. Future research should assess guardian attitudes and preferences for actual trial protocols, as they are developed; using such data to inform protocols is consistent with patient‐focused medical product development.

## 5 CONCLUSIONS

Our study addresses a gap in the literature regarding the willingness of caregivers to allow their children living with HIV to participate in HIV remission trials with ATI. We found primarily positive intentions to enrol and positive attitudes among caregivers of children likely to be eligible for these trials, which supports the feasibility of future paediatric remission trials. Of note, some caregivers reported overly optimistic expectations of trial benefits. While it is important to preserve hope, care must be taken to ensure that caregivers understand that their children may not benefit and that there are risks and burdens of trial participation. Our study identified incomplete disclosure about the child's HIV status as a challenge to informed consent and assent processes. The results inform key areas in which educational and counselling materials will need to be strengthened to ensure ethical and authentic informed consent (and assent, where age‐appropriate) for paediatric remission trials.

## COMPETING INTERESTS

All of the authors declare that their institutions were supported via the EPIICAL consortium funded by an unrestricted grant from VIIV Healthcare to the PENTA‐ID Foundation.

## AUTHOR CONTRIBUTIONS

LK, HLP, SB and KO: Conceptualization. KO: Data curation. LK and AB‐T: Formal analysis. LK, HLP, MGL, TN, TP, A‐IM, AV, PP and MCI: Funding acquisition. KO, SB, MGL, TN, DR, TP, MA, AM, AV and MC: Investigation. HLP: Methodology. AB‐T: Visualization. LK, HLP and ABT: Writing—original draft. All authors: Writing—review and editing.

## FUNDING

This work is part of the EPIICAL project (http://www.epiical.org/), supported by PENTA‐ID foundation (http://penta‐id.org/), funded through an independent grant by ViiV Healthcare, United Kingdom. The work was also supported by the Eunice Kennedy Shriver National Institutes of Child Health and Human Development grant HD115480.

## Data Availability

The data that support the findings of this study are available from the corresponding author upon reasonable request.
